# Comparison of indigo carmine decolorization by Pseudomonas aeruginosa and crude laccase enzyme from Funalia trogii

**DOI:** 10.3906/biy-1807-48

**Published:** 2019-02-07

**Authors:** Filiz BORAN, Emre BİRHANLI, Özfer YEŞİLADA, Elif ÖZBEY

**Affiliations:** 1 Department of Biology, Faculty of Arts and Sciences, İnönü University , Malatya , Turkey

**Keywords:** Bacterium, crude laccase, decolorization, indigo carmine

## Abstract

The effects of incubation time, temperature, initial pH, and dye concentration on the indigo carmine decolorization activity of Pseudomonas aeruginosa ATCC 10145 and some factors on the decolorization potential of crude laccase enzyme obtained from Funalia trogii ATCC 200800 were comparatively investigated. This bacterium showed effective decolorization activity at all agitation and temperature values. Indigo carmine was greatly decolorized by P. aeruginosa at all pH values except pH 10. A decrease in decolorization activity occurred with increasing dye concentration, but this bacterium effectively decolorized the dye within 24 h. The decolorization process was through microbial metabolism, not biosorption. No decolorization or laccase activity could be obtained by the cell-free intracellular extract or culture filtrate of this bacterium. On the other hand, crude laccase effectively decolorized indigo carmine under highly acidic conditions, especially at pH 3.0 as 57% in 300 seconds. This activity decreased progressively due to the increase in pH values. In a short incubation period and at high temperature values, the crude laccase enzyme removed the color of the dye at 50 °C (56%), 60 °C (45%), and 70 °C (38%). These data are important for improving methods for decolorization of textile dyes used at high temperatures in various industrial applications.

## 1. Introduction

Textile dyes are the main pollutants in the textile and dyeing industry’s wastewater. Approximately 5%–10% of the dyes used are released into the environment with wastewater, and the colored wastewater negatively affects photosynthetic activity and dissolved oxygen concentration in water bodies into which it is released. Therefore, the decolorization of this type of wastewater is generally more important than the remediation of the other colorless organic substances (Banat et al., 1996; Wong and Yu, 1999) . Generally, textile dyes are highly recalcitrant to biological degradation. Thus, textile and dyeing industry wastewater is not effectively decolorized by conventional biological treatment systems such as activated sludge systems. There have been many studies on decolorization of wastewater containing dyes using various methods and biological systems (Yesilada et al., 2003; Barka et al., 2008; Ramya et al., 2008; Manivannan et al., 2011) .

Wastewater with dyes may be decolorized using various biological systems such as fungi, enzymes, and bacteria (Campos et al., 2001; Yesilada et al., 2010; Kalyani et al., 2012; Wang et al., 2012; Yeşilada et al., 2014a) . Indigo dye (C.I. 73015 Acid Blue 74) is used to dye denim fabric (Ramya et al., 2008) . Its toxicity has also been reported (Barka et al., 2008) . Because it is recalcitrant to activated sludge system decolorization, high amounts of indigo dye are released into rivers and lakes with wastewater. Due to its negative effects, it must be decolorized using ecofriendly methods. Bacterial, fungal, and enzymatic decolorization of indigo carmine has also been reported (Barka et al., 2008; Ramya et al., 2008; Birhanli and Yesilada, 2010; Terres et al., 2014) . The dye decolorization performance of bacteria and laccase enzymes are different and they need different optimum decolorization conditions for optimum levels of decolorization. Although there have been several studies on decolorization activity of bacteria and laccase enzyme, based on our literature knowledge, there have been no studies that focus on the comparison of indigo dye decolorization using a bacterium and crude laccase enzyme from white rot fungus Funalia (Trametes) trogii ATCC 200800. Therefore, in this study, the indigo dye decolorization activity of Pseudomonas aeruginosa and the crude laccase obtained from the white rot fungus Funalia trogii ATCC 200800 under the effects of various culture conditions was comparatively investigated.

## 2. Materials and methods

### 2.1. Textile dye

Indigo carmine (Acid blue 74) was prepared as a stock solution of 1000 mg/L by dissolving in distilled water and utilized at various concentrations (50–500 mg/L).

### 2.2. Bacterium and culture conditions

Pseudomonas aeruginosa ATCC 10145 was tested for its dye decolorization activity. This bacterium was first incubated at 30 °C on Luria agar (LA) plates. A loopful of P. aeruginosa culture was then inoculated into 20 mL Luria broth (LB)/100 mL Erlenmeyer flask and incubated at 30 °C and 150 rpm. After incubation, an aliquot of 1 mL overnight culture was inoculated into a 100-mL flask containing 20 mL of LB and cultured at 30 °C and 150 rpm for 24 h of incubation. As the final step, 1 mL of this culture was transferred into 250-mL Erlenmeyer flasks with 50 mL LB containing textile dye.

The effects of agitation, temperature, pH, dye concentration, and culture period on the indigo carmine decolorization ability of P. aeruginosa was studied. Unless otherwise stated, the agitation and temperature values were 150 rpm and 30 °C, respectively. Flasks containing only dye and medium but no bacteria were used as controls.

### 2.3. Bacterial decolorization studies

The effect of incubation time on the dye decolorization activity of P. aeruginosa was tested for 2, 4, and 6 h under static and shaking (150 rpm) conditions. The effect of pH on decolorization was tested within the pH range of 5.0–10.0. Dye decolorization potential of the bacterium was tested under static and various agitated conditions at 50–200 rpm after 4 h incubation. In order to detect the effect of incubation temperature on decolorization activity, different temperature values (20–50 °C) were used. To test the effects of initial dye concentration, the bacterium was treated with the dye solutions of 100–500 mg/L concentrations for 4, 8, and 24 h.

### 2.4. Live and dead bacterial pellet studies

The dye decolorization potential of live and dead microbial pellets was also compared. To examine the decolorization activity of live and dead P. aeruginosa pellets, P. aeruginosa was cultivated in LB without indigo carmine dye as stated above; the culture was then centrifuged at 6000 rpm. After centrifugation, the pellets were autoclaved at 121 °C for half an hour for preparation of dead pellets. Live pellets were also prepared in the same manner, but without autoclavization. The prepared dead and live pellets were added separately into 50 mL LB containing 50 and 100 mg/L indigo carmine dye and incubated for 4 h.

### 2.5. Dye decolorization activity of cell-free culture filtrate and cell-free intracellular extract of the bacterium

To test if the decolorization activity was from cell-free culture broth, P. aeruginosa culture filtrate obtained after indigo carmine dye decolorization for 4 h was used. This decolorized culture broth was filtered with a sterilized membrane of 0.45-mm pore size, and then 50 mg/L indigo carmine (at final concentration) was added to this cell-free culture filtrate and incubated at 30 °C and 150 rpm for 4 h.

To determine if this bacterial dye decolorization activity was from cell-free intracellular extract, 4 mL phosphate buffer (pH 4.0) was added to the bacterial pellets from an overnight culture centrifuged at 6000 rpm, and sonicated with an ultrasonic processor. The cell-free intracellular extract was then obtained by centrifugation at 10000 ×g and 4 °C for 10 min.

The presence of laccase enzyme activity in the cellfree culture filtrate and cell-free intracellular extract was also investigated. The laccase production ability of this bacterium was also screened by using a nutrient agar plate containing 0.5 mM ABTS. This plate was incubated at 30 °C for 48 h after inoculation from the P. aeruginosa overnight culture. The oxidation of ABTS indicates laccase production.

### 2.6. Crude laccase enzyme production by Funalia trogii

The preinoculum was obtained by incubating the mycelium of Funalia trogii ATCC 200800 in 100 mL of Sabouraud dextrose broth (SDB) at 30 °C and 150 rpm for 5 days. The formed preinoculum was homogenized, and 7 mL of this homogenate was transferred to 600 mL of fresh SDB and then incubated for 5 days. The obtained pellets were used for the production of laccase enzyme with the repeated-batch method. During this process, 50 mL of the stock basal medium (SBM) were added to the pellets and they were incubated for 24 h under agitated conditions. The composition of SBM utilized was (g/L): KH2PO4, 0.2; CaCl2.2H2O, 0.1; MgSO4.7H2O, 0.05; NH4H2PO4, 0.5; FeSO4.7H2O, 0.035; glucose, 2; yeast extract, 1; CuSO4.5H2O, 0.125. Every 24 h for 5 days, SBM was completely removed and fresh medium added to the filtered pellets (Birhanli and Yesilada, 2010) . The culture filtrate from the 5 days of incubation was used as the source of crude laccase for color removal studies. The crude laccase was added into the solution containing indigo carmine (100 mg/L) and citrate phosphate buffer and incubated for 30, 60, and 300 s. The impact of pH, incubation temperature, and amounts of crude enzyme on dye decolorization was tested for all incubation times as mentioned above. Unless otherwise stated, the incubation temperature, dye, and amount of crude enzyme were 30 °C, 100 mg/L, and 100 µL, respectively.

### 2.7. Decolorization studies with the crude laccase enzyme from F. trogii

The effect of pH on decolorization was tested within the pH range of 2.5–6.0 for crude laccase. Citrate phosphate buffer was used for adjusting the pH values of the reaction mixtures. A temperature range of 30–70 °C was used for testing the dye decolorization activity of the crude laccase at pH 3.0. Different amounts (10–200 µL) of crude laccase enzyme were used to detect the impact of the enzyme amount on dye decolorization. All of the decolorization studies with crude fungal laccase were carried out at 100 mg/L dye concentration and 150 rpm.

### 2.8. Assays

Dye decolorization was calculated by monitoring the absorbance changes at the maximum absorbance wavelengths (610 nm) of indigo carmine. The decolorization values were expressed in terms of percentage.

Laccase (EC 1.10.3.2) activity was determined by measuring the increase in absorbance of ABTS [2,2′-Azinobis(3-ethylbenzthiazoline-6-sulfonic acid)] at 420 nm ( Yesilada et al., 2014 b). The assay mixture contained 100 mM sodium acetate buffer (pH 5.0), 0.5 mM ABTS, suitable amounts of cell-free culture filtrate and cell-free intracellular extract of the bacterium, and culture filtrate of F. trogii (Birhanli and Yesilada, 2006; Birhanli et al., 2013) . All values are the means of at least 3 replicates.

## 3. Results and discussion

### 3.1. Dye decolorization by P. aeruginosa

Because physical and chemical methods have various disadvantages for dye removal, various bacteria, as biological systems, have been studied for the decolorization of textile dyes such as indigo carmine (Table 1).

**Table 1:** Decolorization of indigo carmine by different bacteria.

Bacteria	T (°C)	pH	DC (mg/L)	DT	DR (%)	Reference
Bacillus sp. MZS10	30	7.0	100	15 h	87	Li et al. (2015)
Citrobacter amalonaticus Y19	35	-	50	48 h	12.5	Oh et al. (2011)
Bacillus sp.	37	6.0	100	96 h	66.6	Jaiswal et al. (2014)
Paenibacillus larvae	30	7.0	100	8 h	100	Ramya et al. (2008)
Aeromonas hydrophila DEC1	30	7.0	100	24 h	60	Chen et al. (2003)
Aeromonas hydrophila DEC2	30	7.0	100	24 h	50	
Aeromonas hydrophila DEC3	30	7.0	100	24 h	46	
Aeromonas hydrophila DEC4	30	7.0	100	24 h	40	
Aeromonas hydrophila DEC5	30	7.0	100	24 h	30	
Aeromonas hydrophila DEC6	30	7.0	100	24 h	26	
Pseudomonas sp. GM3	35	7.0	100	48 h	69	Yu et al. (2001)
Pseudomonas sp. Q3	35	7.0	100	48 h	61	
Pseudomonas sp. Z1	35	7.0	100	48 h	88	
Pseudomonas aeruginosa	30	6.0	50	4 h	92	This study
	30	6.0	100	4 h	91	

*T: Temperature, DC: Dye concentration, DT: Decolorization time, DR: Decolorization rate.

### 3.1.1. The effect of incubation time on biodecolorization

**Figure 1:**
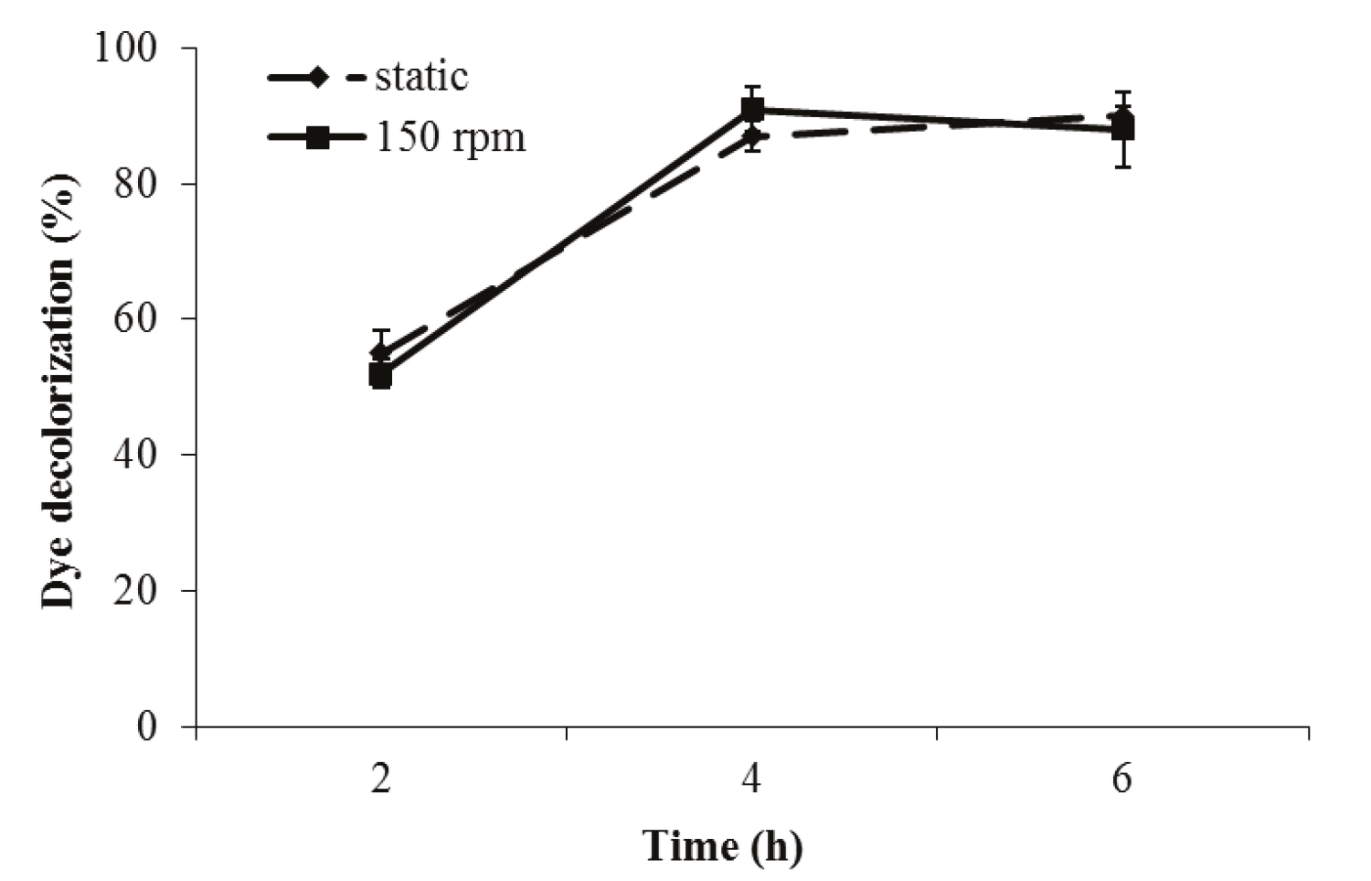
Effect of incubation time on indigo carmine dye (50 mg/L) decolorization activity of P. aeruginosa.

**Figure 2:**
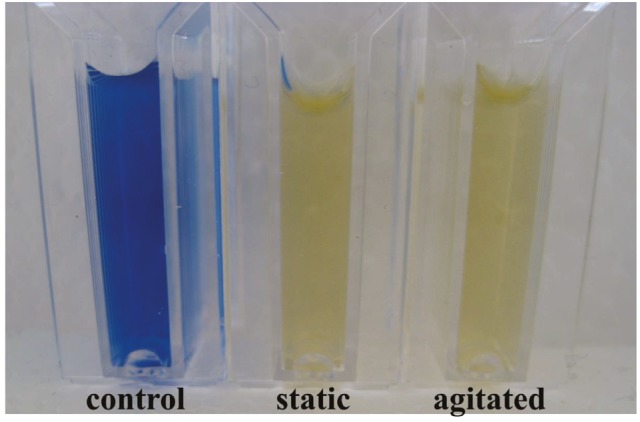
Indigo carmine (50 mg/L) decolorization of P. aeruginosa after 4 h of incubation under static and agitated conditions.

To test the effect of incubation time on biodecolorization of indigo carmine dye, the bacterium was incubated in 50 mg/L dye-containing medium under static and agitated (150 rpm) conditions for 6 h. The bacterium showed rapid decolorization activity; it effectively decolorized this dye after 4 h of incubation. The decolorization value was not significantly changed after this time (Figure 1). Therefore, incubation for 4 h was determined as a suficient incubation period for testing the indigo carmine dye decolorization activity of this bacterium. Chen et al. (2003) used 6 different isolates of Aeromonas hydrophila for decolorization of indigo carmine dye at 100 mg/mL concentration. The maximum decolorization activity was obtained in the first tested isolate as 60% and 84% at the first and seventh days of incubation, respectively. Decolorization of indigo carmine (50 mg/L) under static and agitated conditions is shown in Figure 2.

### 3.1.2. The effect of agitation on biodecolorization

**Figure 3:**
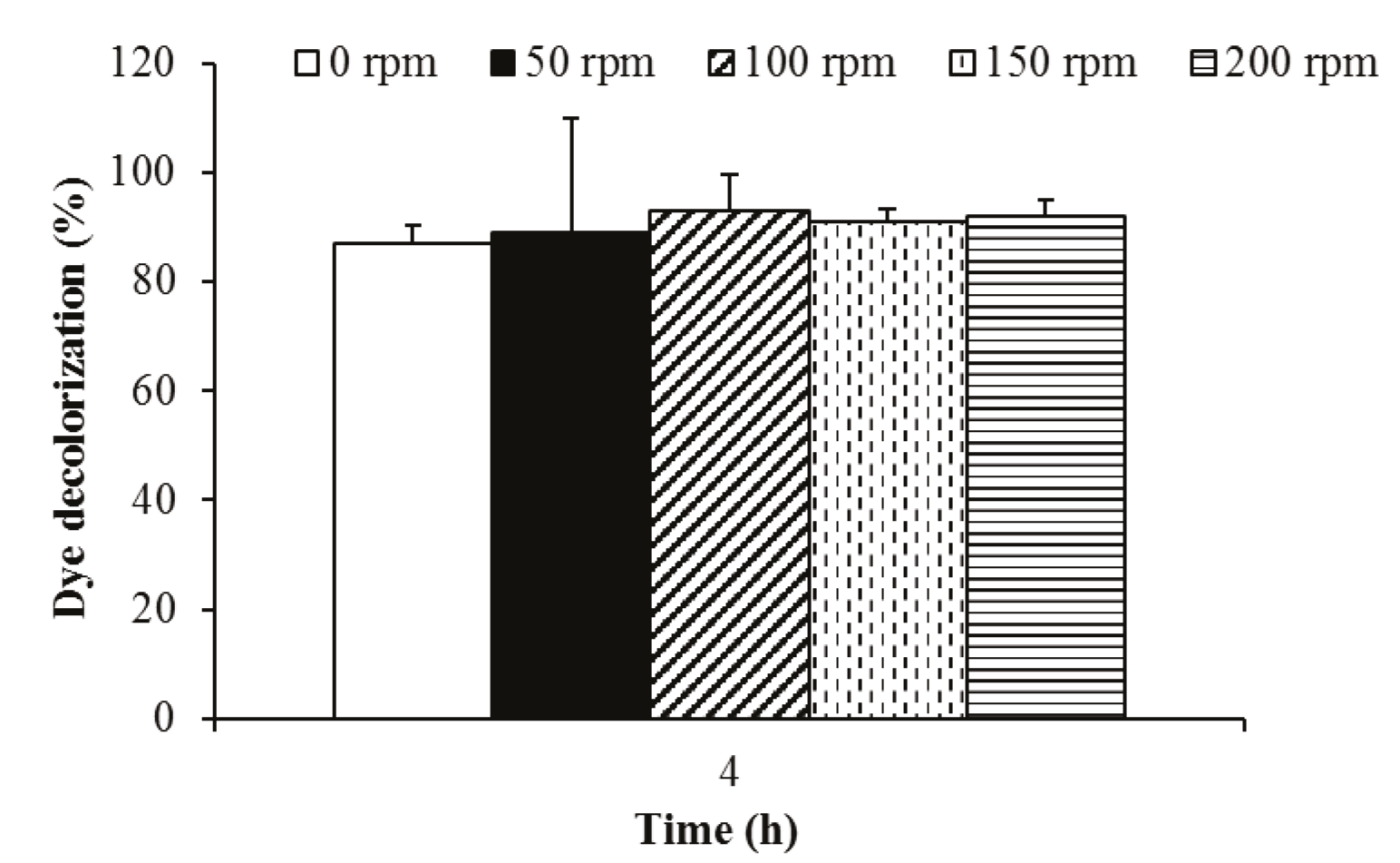
Effect of agitation on indigo carmine dye (50 mg/L) decolorization activity of P. aeruginosa.

Agitation is an important factor for high decolorization activity. Therefore, the effect of agitation on the dye decolorization ability of P. aeruginosa was also investigated. To this end, dye decolorization ability of the bacterium was tested under static and various agitated conditions. This bacterium effectively decolorized this dye at all agitation rates after 4 h of incubation (Figure 3). Ramya et al. (2008) reported low indigo dye decolorization activity This study with nonshaken cultures of Paenibacillus larvae compared to shaken cultures. Similarly, free cells of Pseudomonas luteola were reported as very sensitive to dissolved oxygen during reactive red 22 dye decolorization (Chang et al., 2001) . This difference may come from the different species used.

### 3.1.3. The effect of temperature on biodecolorization

**Figure 4:**
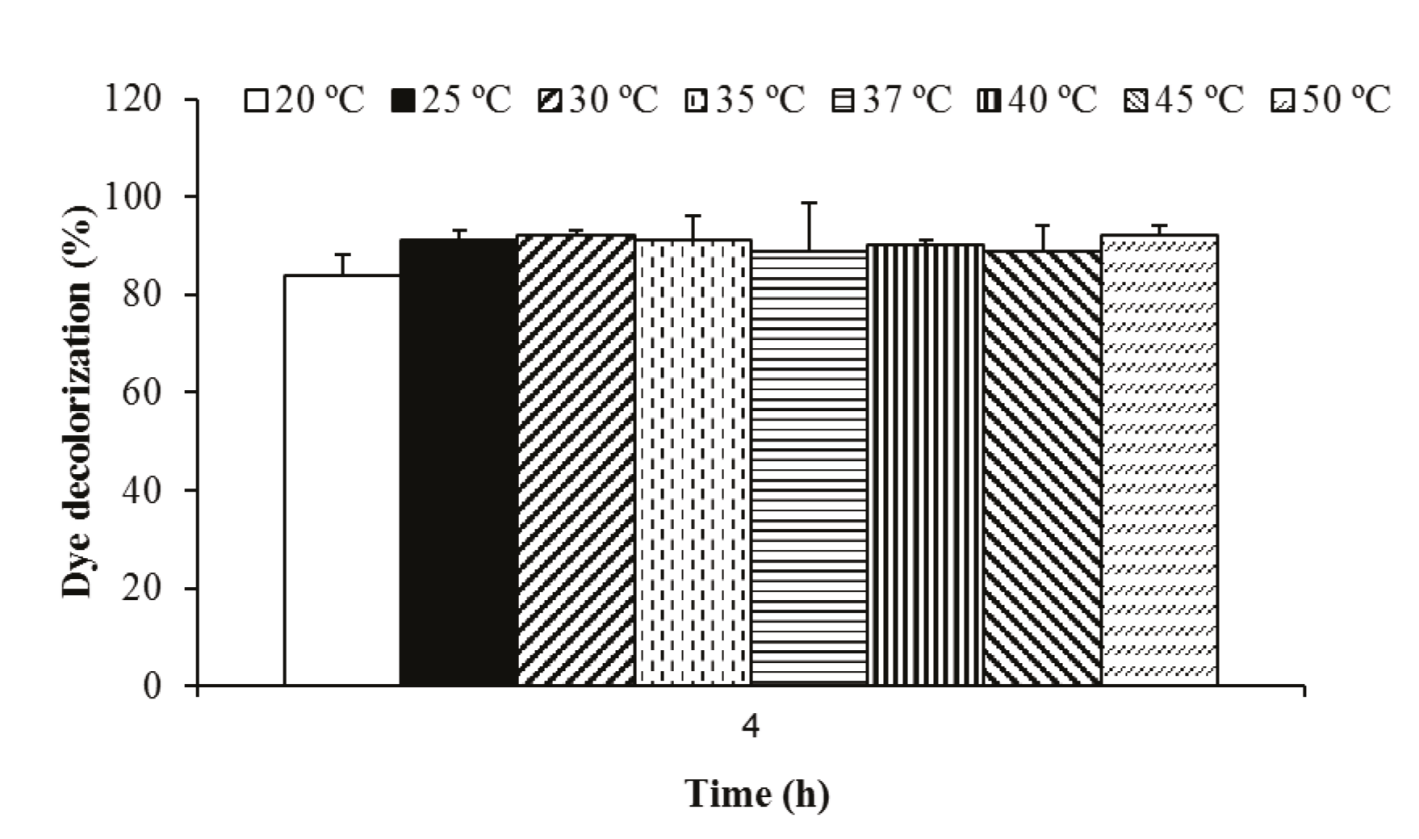
Effect of temperature on indigo carmine dye (50 mg/L) decolorization activity of P. aeruginosa.

Decolorization of indigo carmine dye was carried out at various temperatures (20–50 °C) at 150 rpm for 4 h. Figure 4 shows that P. aeruginosa decolorized this dye eficiently at all of the temperatures tested. This showed that dye decolorization activity of P. aeruginosa is independent of temperature changes. Ramya et al. (2008) reported low indigo dye decolorization activity of bacterium Paenibacillus larvae at a temperature of 20 °C. The indigo carmine decolorization capacity of this strain under a broad range of temperatures shows that this strain could be effectively used in dye decolorization. Jaiswal et al. (2014) investigated various reaction parameters such as incubation temperature (20, 33, and 37 °C), pH (5.0–10.0), and incubation time (24–96 h) in order to find the highest decolorization rate of indigo carmine (100 mg/L) using Bacillus sp. The maximum decolorization activity was observed as about 67% at pH 6.0 and 37 °C under agitated conditions (120 rpm) in the 96th h.

### 3.1.4. The effect of pH on biodecolorization

**Figure 5:**
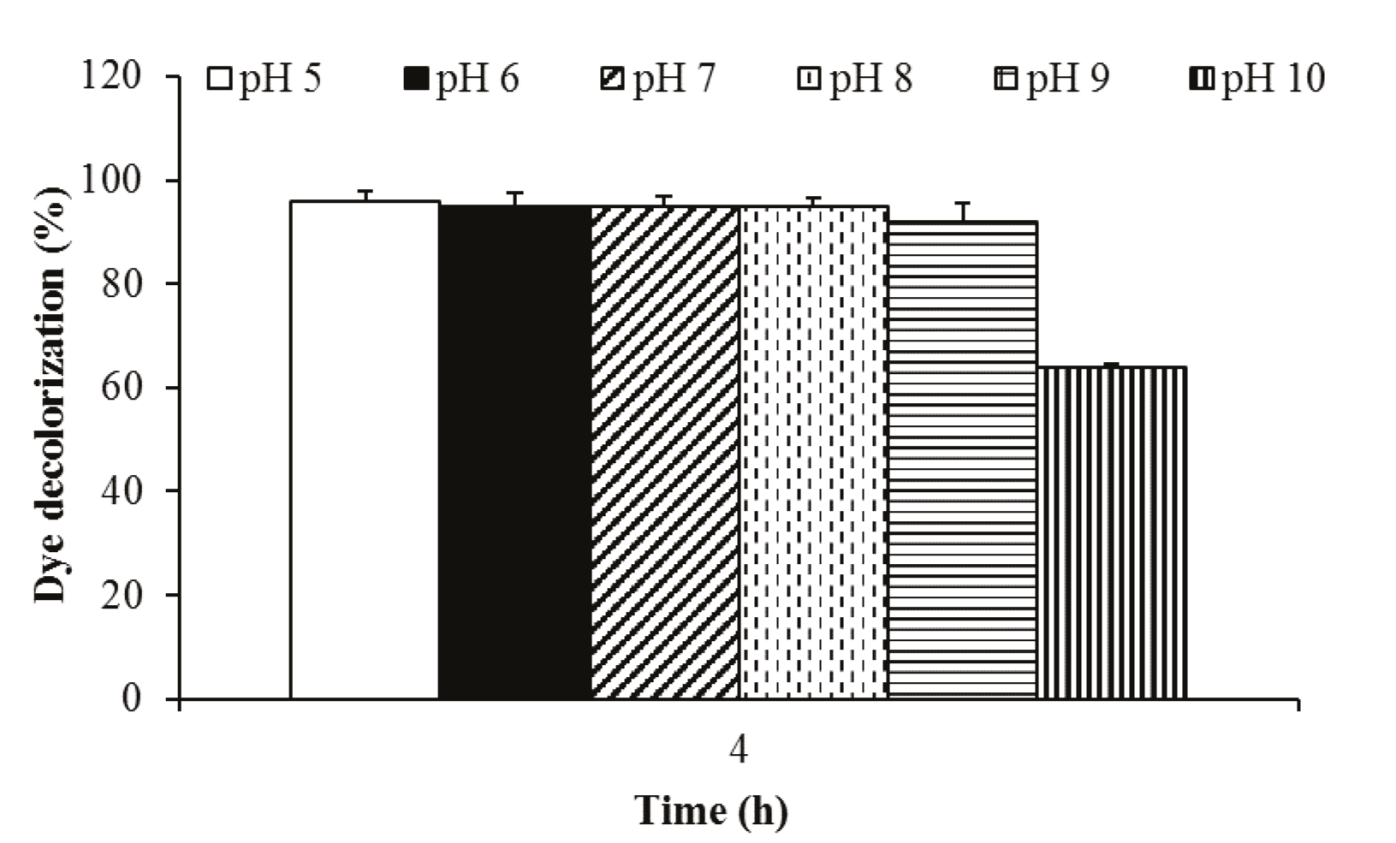
Effect of pH on indigo carmine dye (50 mg/L) decolorization activity of P. aeruginosa.

The pH of the medium may affect the dye decolorization activity of bacteria (Hsueh and Chen, 2007) . Therefore, the effect of pH on the dye decolorization activity of this bacterium was also investigated at different initial pH values (150 rpm and 30 °C). As shown in Figure 5, this bacterium could highly decolorize this dye at all pH values except pH 10.0. This bacterium could be used to decolorize dye wastewater of acidic, neutral, and basic types. The decolorization of dyes at a wide range of pH values is a desirable characteristic (Kalyani et al., 2012) . Ramya et al. (2008) reported low indigo dye decolorization activity (6%–15%) of bacterium Paenibacillus larvae at pH 3.0, while the maximum decolorization activity was at pH 7.0 and 8.0.

### 3.1.5. The effect of initial dye concentration on biodecolorization

**Figure 6:**
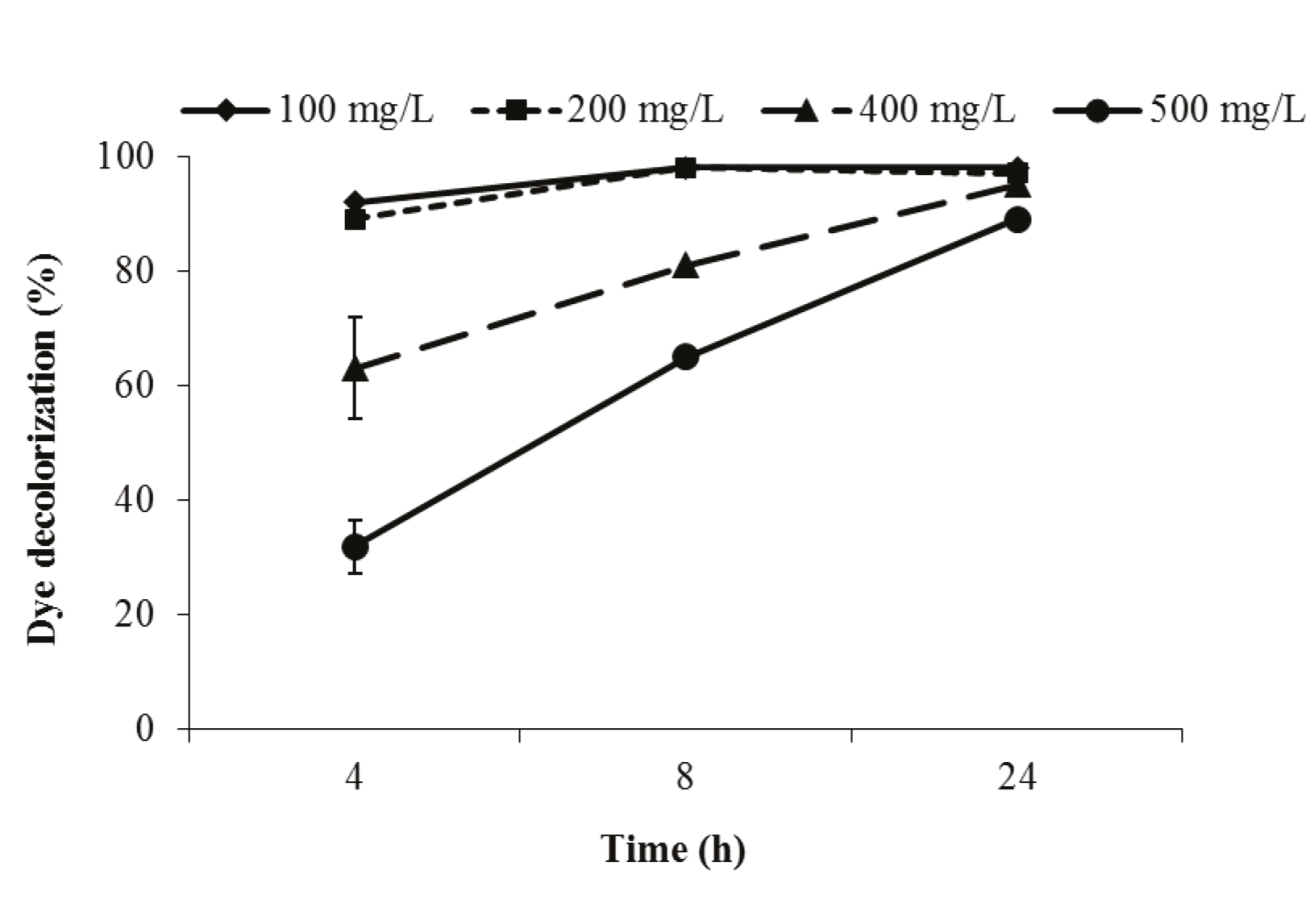
Effect of different initial dye concentrations on indigo carmine dye decolorization activity of P. aeruginosa.

Indigo carmine decolorization activity of P. aeruginosa was also tested for different initial dye concentrations (100–500 mg/L) at 30 °C and 150 rpm. An increase in dye concentration negatively affected short-duration decolorization activity, and dye decolorization activity of this bacterium decreased with increasing dye concentration. However, the bacterium recovered its decolorization activity for longer incubation times. Therefore, high dye concentrations were not toxic to P. aeruginosa. Similar results were reported by Ramya et al. (2008) . P. aeruginosa effectively decolorized this dye within 24 h at all concentrations used (Figure 6). Controls containing dyes but with no cells showed no change in color.

### 3.1.6. Indigo carmine dye decolorization under static and agitated conditions

The dye decolorization activity of this bacterium was compared under static and agitated conditions over the course of 6 h. The dye decolorization activity was studied at pH 6.0, dye concentration of 50 mg/L, and 2 different temperatures (30 and 40 °C). The observed dye decolorization activity of this bacterium under static and agitated conditions was quite high (Table 2).

**Table 2:** Indigo carmine dye decolorization activity of P. aeruginosa under static and agitated conditions.

	Dye decolorization (%)
Time (h)	30 °C	40 °C
	Static	Agitated	Static	Agitated
2	59 ± 5.29	59 ± 8.39	72 ± 4.58	89 ± 2.00
4	87 ± 1.73	92 ± 1.00	90 ± 1.00	93 ± 0.58
6	86 ± 4.51	92 ± 1.53	91 ± 0.58	93 ± 0.58

### 3.1.7. Indigo carmine dye decolorization activity of dead and live pellets of P. aeruginosa

Biodecolorization is due to either adsorption or biodegradative decolorization. Therefore, the indigo carmine dye decolorization ability of dead and live pellets of P. aeruginosa was also compared to prove that the dye decolorization activity is from metabolic activity of this bacterium, and not from adsorption. This decolorization activity was tested for 2 different dye concentrations (50 mg/L and 100 mg/L) at 30 °C and 150 rpm. While the dead pellets decolorized 20%–27% of the dye, live pellets rapidly decolorized 91%–92% of the dye without any visible adsorption (Table 3). Therefore, the high percentage of dye decolorization is mainly due to bacterial metabolism, not adsorption. This result is similar to those observed with other Pseudomonas strains (Yu et al., 2001) . Malachite green dye decolorization activity of the P. aeruginosa NCIM 2074 strain was attributed to biodegradation and not adsorption (Kalyani et al., 2012) .

**Table 3:** Indigo carmine dye decolorization ability of dead and live pellets of P. aeruginosa.

	Dye decolorization (%)
Time (h)	50 mg/L	100 mg/L
	Dead pellets	Live pellets	Dead pellets	Live pellets
1	15 ± 1.53	91 ± 2.65	27 ± 4.04	89 ± 0.58
2	16 ± 3.06	91 ± 3.00	27 ± 1.53	91 ± 0.00
3	16 ± 4.51	88 ± 4.51	28 ± 2.00	91 ± 0.00
4	20 ± 3.06	92 ± 2.65	27 ± 3.06	91 ± 0.58

### 3.1.8. Dye decolorization activity of cell-free intracellular extract and culture filtrate

It is possible to decolorize the dyes by using cell-free intracellular extract or extracellular culture filtrates of various organisms ( Yeşilada et al., 2014 a). Therefore, the decolorization activity of cell-free intracellular extract and culture filtrate was also tested. However, in this study, no decolorization could be obtained with either the cell-free intracellular extract or culture filtrate of this bacterium. Kuddus et al. (2013) reported the textile dye decolorization ability of culture filtrate of P. putida .

The laccase enzymes from fungi or bacteria have dye decolorization activity (Reiss et al., 2011) . The indigo dye decolorization activity of laccase from bacteria has also been reported (Cho et al., 2011; Reiss et al., 2011) . Therefore, the laccase production ability of this bacterium was screened by using nutrient agar plate containing 0.5 mM ABTS. Because ABTS is a substrate for laccase, the colored oxidation product can be seen macroscopically if this bacterium produces this enzyme. Absence of oxidation indicated that there was no laccase production. After this preliminary study, we assayed the laccase activity of cellfree intracellular extract and extracellular culture filtrate spectrophotometrically. No laccase activity was detected. Yu et al. (2001) reported a nonspecific decomposition mechanism for decolorization of indigo carmine by Pseudomonas strains.

### 3.2. Dye decolorization by crude laccase

Laccase, an ecofriendly enzyme, can be used for various industrial applications. But it is important that the production of this enzyme produces large amounts and be cost effective. This enzyme may be synthesized by different fermentation processes such as submerged and solidstate fermentation. The repeated-batch mode, a different method from the others stated above, was used in this study. Large amounts of laccase were extracellularly synthesized with this method, which used the pellet forms of F. trogii. White rot fungal laccases could decolorize textile dyes such as indigo carmine (Table 4). Therefore, the crude laccase obtained here was used for the decolorization of the commonly used textile dye indigo carmine.

**Table 4:** Decolorization of indigo carmine by different fungal laccases.

Laccase obtained from	T (°C)	pH	DC (mg/L)	DT	DR (%)	Reference
Trametes trogii BAFC 463	30	4.5	23	30 min	94	Levin et al. (2010)
Myceliophthora thermophila	30	5.5	20	16 h	31	Claus et al. (2002)
Polyporus pinisitus	30	5.5	20	16 h	83	
Trametes versicolor	30	5.5	20	16 h	92	
Polyporus pinisitus	30	5.9	200	16 h	86 (with 2 mM HBT)	
Trametes versicolor DSM 11269	50	5.5	62.5	6 h	10	Theerachat et al. (2012)
Trametes modesta	50	4.5	250	6 h	58	Nyanhango et al. (2002)
Trametes sp. SYBC-L4	30	4.5	100	36 h	99 (with 2.5mM HBT)	Li et al. (2014)
Pleurotus sajor-caju	32	5.0	46.6	3 h	≥ 90	Sarnthima and Khammuang (2008)
Ceriporiopsis subvermispora CZ-3	30	7.0	100	24 h	95	Yavuz et al. (2014)
	30	7.0	100	0.5 h	≤ 100 (with 6 mM HBT)	
Funalia (Trametes) trogii ATCC 200800	30	-	100	72 h	87	Birhanli and Yesilada (2006)
Trametes versicolor ATCC 200801	30	-	100	72 h	88	
Funalia (Trametes) trogii ATCC 200800	30	3.0	100	5 min	57	This study

*T: Temperature, DC: Dye concentration, DT: Decolorization time, DR: Decolorization rate.

### 3.2.1. The effect of pH on biodecolorization

The reaction pH is an important parameter for dye decolorization by enzyme. For this aim, different pH values (2.5–6.0) were investigated at 30, 60, and 300 s of incubation time in order to detect the optimum pH value for high decolorization of indigo carmine by crude laccase. Decolorization of indigo carmine dye at 100 mg/L concentration was carried out at 150 rpm and 30 °C. The differences in dye decolorization of fungal laccase depending on the various pH values are presented in Table 5. Some researchers have reported acidic pH values as the optimum pH values for fungal laccases in dye decolorization studies (Li et al., 2014) . In a study conducted by Li et al. (2014) , indigo carmine dye at 100 mg/L was decolorized at a rate of 99% by crude laccase of Trametes sp. SYBC-L4 at an optimum pH of 4.5 within 36 h. Similarly, pH 3.0 was determined as the optimum pH for the decolorization of indigo carmine in this study.

**Table 5:** The effect of different pH values on decolorization of indigo carmine by crude laccase at 30 °C.

	Decolorization (%) of indigo carmine
pH values	30 s	60 s	300 s
2.5	7.78 ± 1.96	14.21 ± 0.70	41.48 ± 2.28
3.0	14.74 ± 1.65	26.36 ± 2.68	57.16 ± 2.50
3.5	11.99 ± 1.64	24.64 ± 4.78	44.12 ± 1.48
4.0	10.40 ± 1.85	16.33 ± 1.57	23.98 ± 1.74
4.5	8.62 ± 1.38	7.88 ± 0.34	16.79 ± 0.71
5.0	5.73 ± 1.36	9.83 ± 1.74	13.88 ± 1.90
6.0	2.46 ± 1.01	4.03 ± 1.05	6.67 ± 0.30

While indigo carmine was enzymatically decolorized at approximately 57% at pH 3.0, the decolorization activity was sharply decreased to 7% at pH 6.0 over 300 s.

### 3.2.2. The effect of temperature on biodecolorization

**Figure 7:**
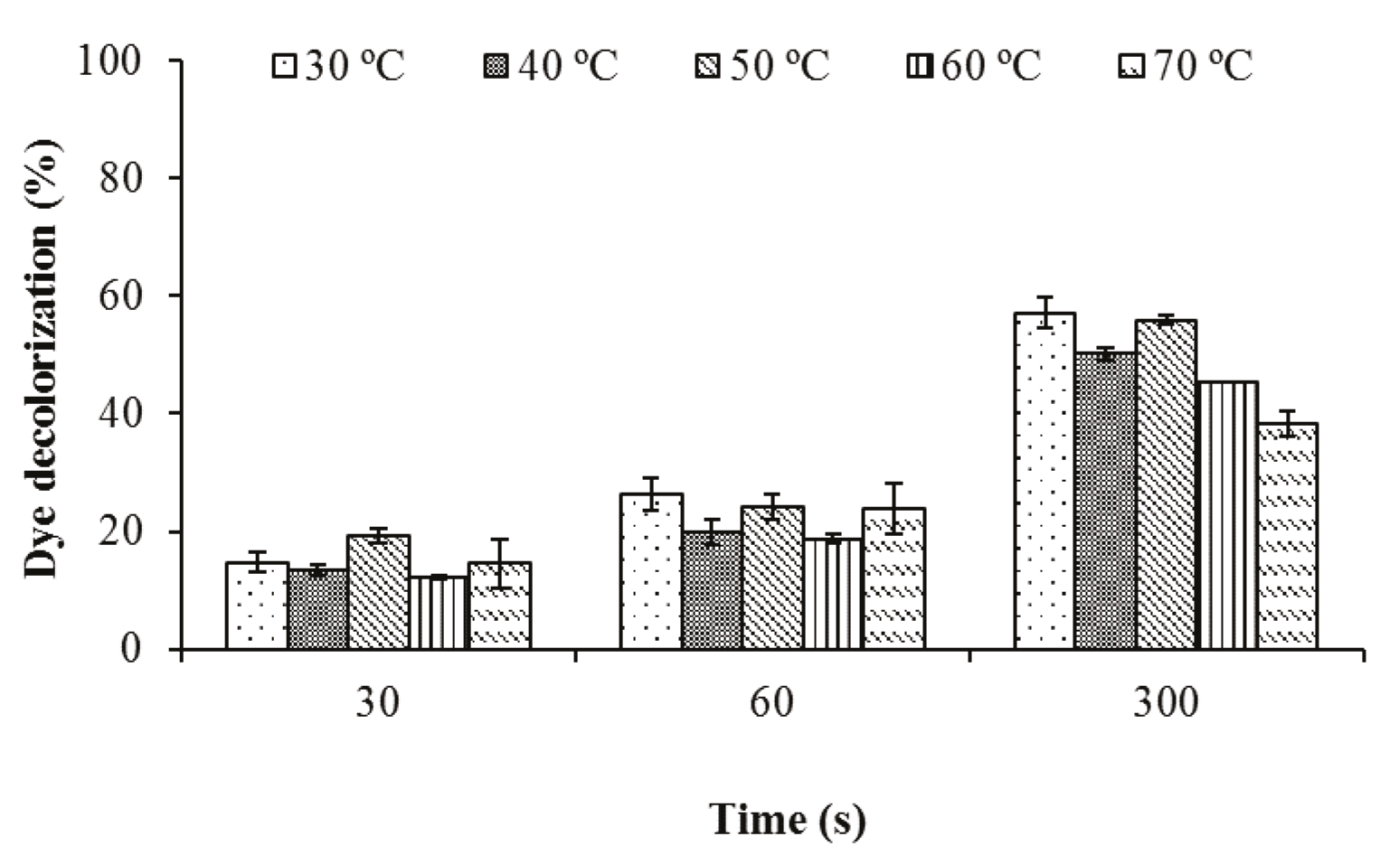
The effect of different temperatures on decolorization of indigo carmine by crude laccase at pH 3.0.

Temperature is a critical parameter for laccase activity ( Yesilada et al., 2014 b) and also for enzymatic decolorization of the textile dye (Yeşilada et al., 2014 a). The decolorization studies at various temperature values between 30 and 70 °C at pH 3.0 gave high decolorization activities. The decolorization values detected at 60 °C and pH 3.0 were 12% and 45% after 30 s and 300 s of incubation, respectively. However, 38% of the color of this dye could be removed at 70 °C and pH 3.0 (Figure 7). In a study performed by Claus et al. (2002) , laccases obtained from Myceliophthora thermophila, Polyporus pinisitus, and Trametes versicolor decolorized the indigo carmine dye (20 mg/L) at 15%, 90%, and 88% rates respectively at 30 °C and pH 5.5 after 16 h incubation.

### 3.2.3. The effect of enzyme amount on biodecolorization

**Figure 8:**
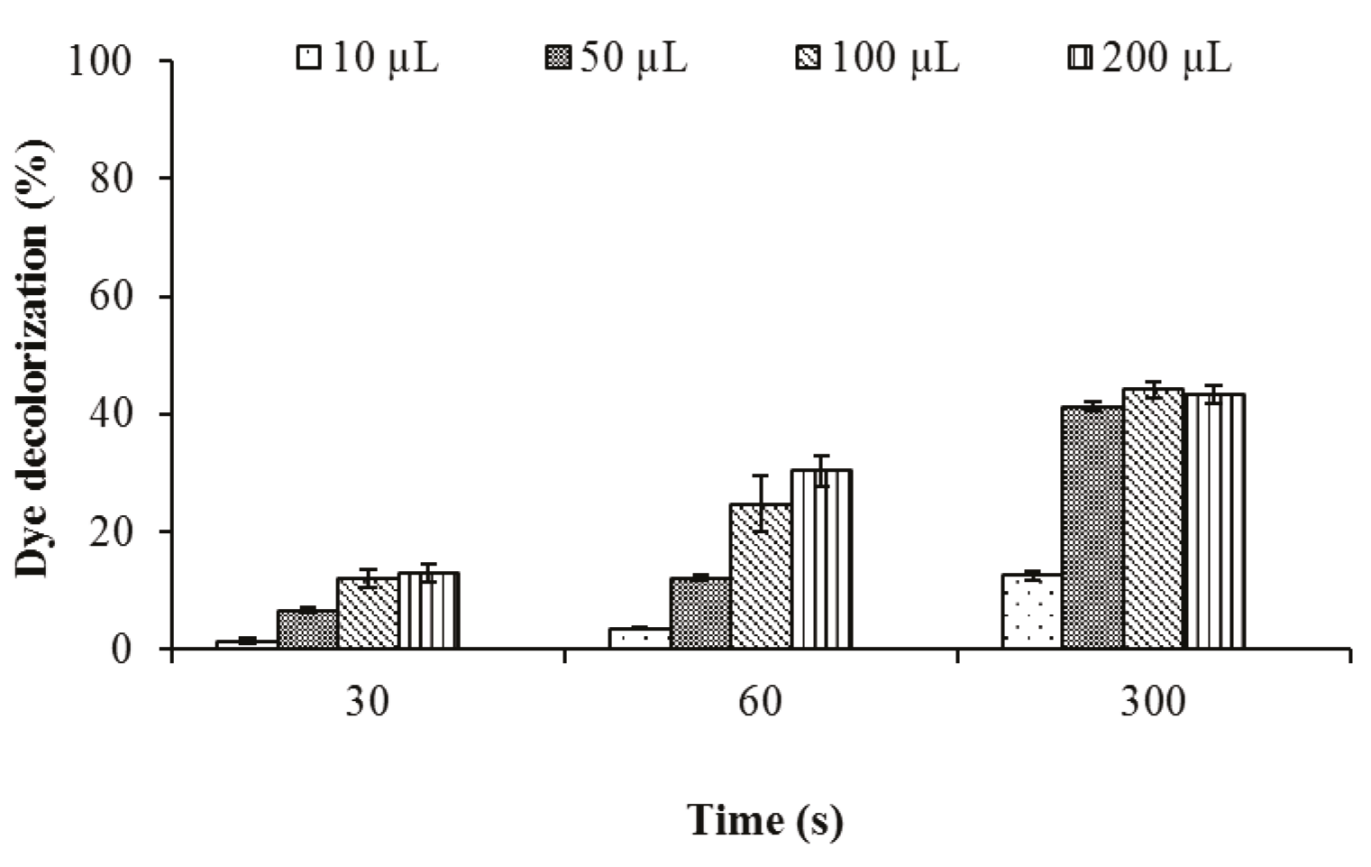
The effect of different amounts of crude laccase on decolorization of indigo carmine at pH 3.0.

The crude laccase (54 U/mL) was utilized to investigate the effect of different amounts of enzyme on indigo carmine biodecolorization. According to the data obtained, the decolorization activity gradually increased with the enzyme amount apart from with 200 µL. As shown in Figure 8, 100 µL was determined as the optimum amount for dye decolorization at pH 3.0.

### 3.3. Conclusion

Indigo carmine is among the most used dyes in textile industry. This dye was greatly decolorized by the bacterium culture used in this study. However, the cell-free intracellular extract or culture filtrate showed no decolorization activity alone. Live pellets rapidly decolorized 91%–92% of the dye but dead pellets decolorized only 20%–27%. This shows that dye decolorization is mainly due to bacterial metabolism. The decolorization of indigo carmine dye by this bacterium was not strongly dependent on the conditions tested. Therefore, this strain could be used to treat textile wastewaters containing indigo dyes. In this work, this dye was also decolorized by crude laccase of F. trogii to various extents depending on the incubation conditions. This crude laccase displayed good decolorization activity over short incubation times, especially at acidic pH values without any mediator. Because some mediators are toxic and expensive, decolorization of the dyes without any mediator may be an ecofriendly and cost-effective method. While the crude laccase decolorized the most dye at pH 3, the bacterium decolorized at all pH values from pH 5 to pH 10. This study showed that bacteria and crude laccase enzyme from fungi should be selected according to the dye type, pH of the wastewater, and type of application. According to the literature, this is the first comparative study on the commonly used textile dye indigo carmine decolorization activity of this bacterium and crude laccase enzyme obtained from F. trogii ATCC 200800. Decolorization processes using bacterial cultures and/or laccase enzyme could be helpful in eliminating environmental pollution due to textile dyes.
